# Skullcapflavone II Suppresses TNF-α/IFN-γ-Induced TARC, MDC, and CTSS Production in HaCaT Cells

**DOI:** 10.3390/ijms22126428

**Published:** 2021-06-16

**Authors:** Hanon Lee, Dong Hun Lee, Jang-Hee Oh, Jin Ho Chung

**Affiliations:** 1 Department of Biomedical Sciences, Seoul National University Graduate School, Seoul 03080, Korea; hanon90@snu.ac.kr; 2 Department of Dermatology, Seoul National University College of Medicine, Seoul 03080, Korea; ivymed27@snu.ac.kr; 3 Institute of Human-Environment Interface Biology, Medical Research Center, Seoul National University, Seoul 03080, Korea; 4 Laboratory of Cutaneous Aging Research, Biomedical Research Institute, Seoul National University Hospital, Seoul 03080, Korea

**Keywords:** Skullcapflavone II, TARC, MDC, CTSS, STAT1, NF-κB, p38 MAPK, anti-inflammatory activity, atopic dermatitis, keratinocytes

## Abstract

Skullcapflavone II (SFII), a flavonoid derived from *Scutellaria baicalensis*, has been reported to have anti-inflammatory properties. However, its therapeutic potential for skin inflammatory diseases and its mechanism are unknown. Therefore, this study aimed to investigate the effect of SFII on TNF-α/IFN-γ-induced atopic dermatitis (AD)-associated cytokines, such as thymus- and activation-regulated chemokine (TARC) and macrophage-derived chemokine (MDC). Co-stimulation with TNF-α/IFN-γ in HaCaT cells is a well-established model for induction of pro-inflammatory cytokines. We treated cells with SFII prior to TNF-α/IFN-γ-stimulation and confirmed that it significantly inhibited TARC and MDC expression at the mRNA and protein levels. Additionally, SFII also inhibited the expression of cathepsin S (CTSS), which is associated with itching in patients with AD. Using specific inhibitors, we demonstrated that STAT1, NF-κB, and p38 MAPK mediate TNF-α/IFN-γ-induced TARC and MDC, as well as CTSS expression. Finally, we confirmed that SFII significantly suppressed TNF-α/IFN-γ-induced phosphorylation of STAT1, NF-κB, and p38 MAPK. Taken together, our study indicates that SFII inhibits TNF-α/IFN-γ-induced TARC, MDC, and CTSS expression by regulating STAT1, NF-κB, and p38 MAPK signaling pathways.

## 1. Introduction

Atopic dermatitis (AD) is a common chronic inflammatory skin disorder characterized by eczematous lesions and intense itch [1]. The pathophysiology of AD involves a variety of genetic, immunological, and environmental factors, which contribute to barrier dysfunction, inflammation, pruritus, and their complex interactions. [2,3]. An important functions of the skin is to serve as a barrier of our body from external stimuli [2]. AD skin lesions have been associated with decreased levels of important components of the skin barrier including filaggrin, loricrin, involucrin, and total ceramides [2]. Following epidermal barrier disruption, keratinocytes produce cytokines such as thymus- and activation-regulated chemokine (TARC), macrophage-derived chemokine (MDC), thymic stromal lymphopoietin (TSLP), interleukin (IL)-25, and IL-33, which recruit many immune cells to the inflamed skin [4]. Most of these are T-helper Th2 cells, which express IL-4 and IL-13 to promote a Th2-related immune response, and are considered a key factor in the pathogenesis of AD [2]. Dendritic cells and other dermal cells activated by IL-4 and IL-13 produce type 2 chemokines, such as TARC, MDC, and CCL18, that attract Th2 cells [2,5,6]. The type 2 related cytokines, IL-4 and IL-13, downregulate the expression of filaggrin and loricrin, exacerbating skin barrier disruption [2]. In addition, IL-4, IL-13, and IL-31 activate downstream Janus kinase (JAK) pathways and induce pruritus [2]. Although the pathogenesis of atopic itch remains unclear, several additional factors related to pruritus, such as histamine-related neuroimmune interactions, activation of protease-activated receptors (PAR), *Staphylococcus aureus* microbiome, and IL-25 stimulation by house dust mite sensitization, have been considered [1,2,7,8].

The chemokines TARC and MDC are important for the development of inflammation and are elevated in skin tissue and plasma of patients with AD [6,9,10,11]. They are produced not only by the infiltrated immune cells but also by keratinocytes when activated by Th2-related cytokines [12,13]. Keratinocytes are not classic immune cells; however, when activated, they secrete Th2-related chemokines, stimulating the infiltration of immune cells into inflamed skin areas and causing inflammatory skin disease [14,15]. Th2-related chemokines derived from keratinocytes include TARC, MDC, and RANTES (Regulated on activated normal T-cell expressed and secreted) [4,16]. Stimulation of HaCaT cells, a human keratinocyte cell line, with TNF-α and IFN-γ is the most widely employed model for TARC and MDC induction [17,18,19]. Such stimulation also induces other pro-inflammatory cytokines and chemokines, such as RANTES, interleukin (IL)-6, IL-1β, TNF-α, and IL-8 [12].

Cathepsin S (CTSS) is a cysteine protease that belongs to the family of cathepsin lysosomal enzymes. CTSS has elastase activity and plays a role in major histocompatibility complex (MHC) class II-mediated antigen presentation. Additionally, CTSS is associated with inflammatory processes, including those in atherosclerosis and asthma, and plays a pruritic role in inflammatory skin diseases such as AD by activating protease-activated receptor 2 (PAR2) [20], which is involved in pain and itching signaling [21]. CTSS is not normally expressed in keratinocytes but is upregulated in IFN-γ-stimulated or inflammatory conditions such as AD [22,23]. In addition, CTSS-overexpressing transgenic mice develop skin diseases similar to chronic AD [24].

Skullcapflavone II (SFII; 5,2′-dihydroxy-6,7,8,6′tetramethoxy-flavone) was isolated from the roots of *Scutellaria baicalensis* with more than 40 other flavonoids, including baicalin, baicalein, and wogonin [25,26]. Flavonoids, a class of polyphenolic secondary metabolites, have various medicinal benefits, such as anti-inflammation, anti-oxidation, anti-tumor, cardio-protection, hepatic-protection, and neuroprotection effects [27,28]. SFII has been reported to exhibit anti-inflammatory and antioxidant activities [29]. Furthermore, it was found to inhibit Th2 cytokine production and mast cell histamine release by regulating NF-κB signaling in allergic rhinitis [26], and it attenuated the major pathophysiological features of ovalbumin-induced allergic asthma in a mouse model by regulating TGF-β1/Smad signaling pathways [30]. SFII also inhibited osteoclastogenesis through redox regulation of MAPKs, Src, and CREB [31] and suppressed FBS- or TNF-α-induced MMP-1 expression by regulating ERK/JNK and NF-κB pathways, respectively, in human dermal fibroblasts [32]. However, the effects of SFII on inflammatory responses in various skin inflammatory diseases, including AD, have not been studied yet. In this study, we evaluated the anti-inflammatory effects of SFII on TNF-α/IFN-γ-induced TARC, MDC, and CTSS production in HaCaT cells and investigated its regulatory mechanisms.

## 2. Results

### 2.1. SFII Significantly Suppresses TNF-α/IFN-γ-Induced TARC, MDC, and CTSS Expression

**Figure 1:**
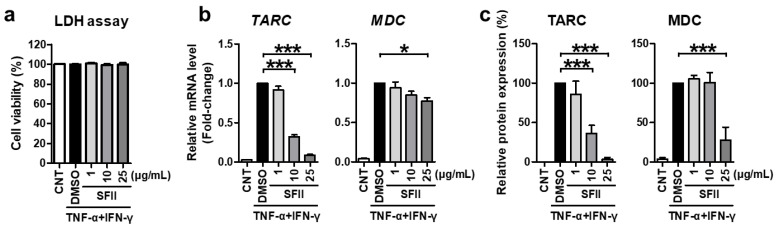
Skullcapflavone II (SFII) significantly suppressed TNF-α/IFN-γ-induced thymus- and activation-regulated chemokine (TARC) and macrophage-derived chemokine (MDC) expression. (**a**) HaCaT cells were treated with 1, 10, and 25 μg/mL of SFII or 0.1% DMSO control (CNT or DMSO labeled) for 24 h, and the LDH assay was performed. For other experiments, cells were stimulated with TNF-α/IFN-γ after SFII pretreatment. (**b**) Then, total RNA was extracted at 18 h, and the mRNA expression of *TARC* and *MDC* was measured by quantitative real-time PCR. (**c**) Conditioned media were harvested 24 h after stimulation, and TARC and MDC protein expression was measured using ELISA. The mRNA and protein levels, relative to those in the TNF-α/IFN-γ-stimulated DMSO control samples, are presented as mean fold-changes ± SEM of independent experiments (N = 4 or 5). Statistical comparisons were performed using a one-way ANOVA followed by Bonferroni’s post-hoc test (* *p* < 0.05, *** *p* < 0.001).

First, we evaluated the cytotoxicity of SFII against HaCaT cells. LDH analysis was performed in the medium, 24 h after SFII or DMSO treatment. SFII did not result in any significant cytotoxicity at the concentrations used in all the experiments in this study (Figure 1a).

Next, we demonstrated the effect of SFII on TARC and MDC. SFII significantly reduced *TARC* and *MDC* mRNA expression induced by TNF-α/IFN-γ (Figure 1b). Furthermore, SFII exerted this inhibitory effect in a dose-dependent manner up to concentrations of 25 μg/mL. TARC and MDC proteins secreted into the culture medium were measured by ELISA. As shown in Figure 1c, the remarkable induction of TARC and MDC by TNF-α/IFN-γ stimulation was significantly suppressed by SFII pretreatment. Consistent with the mRNA data, the inhibitory effect of SFII on TARC was also dose-dependent.

**Figure 2:**
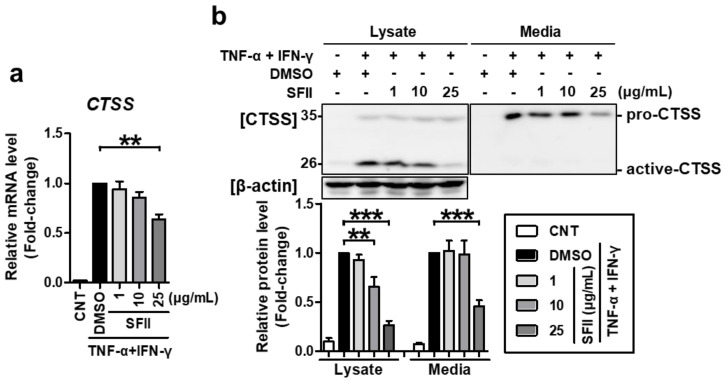
SFII significantly suppressed TNF-α/IFN-γ-induced cathepsin S (CTSS) expression. HaCaT cells were stimulated with TNF-α/IFN-γ after SFII pretreatment. (**a**) Then, total RNA was extracted at 18 h, and the mRNA expression of *CTSS* was measured by quantitative real-time PCR. (**b**) Cell lysates and conditioned media were harvested 24 h after stimulation, and protein levels of CTSS were measured by western blotting. Band intensity was analyzed using ImageJ software, and the combined sum of pro and active CTSS band intensities was normalized to that of β-actin. The mRNA and protein levels, relative to those in the TNF-α/IFN-γ-stimulated DMSO control samples, are presented as mean fold-changes ± SEM of independent experiments (N = 4 or 5). Statistical comparisons were performed using a one-way ANOVA followed by Bonferroni’s post-hoc test (** *p* < 0.01, *** *p* < 0.001).

We then checked whether SFII could also alleviate CTSS expression. *CTSS* mRNA expression was significantly inhibited by 25 μg/mL of SFII treatment (Figure 2a). Using western blotting, CTSS proteins could be detected in two forms, pro-CTSS (35 kDa) and active CTSS (25 kDa). In the lysate, active CTSS was expressed at higher levels than pro-CTSS (Figure 2b). Both forms were significantly increased by TNF-α/IFN-γ-stimulation. However, SFII treatment (10 to 25 μg/mL) significantly suppressed only active CTSS, and not pro-CTSS, in a dose-dependent manner. Meanwhile, in the medium, only pro-CTSS was mainly detected, and active-CTSS was hardly found. Pro-CTSS in the medium was significantly induced by TNF-α/IFN-γ and was significantly inhibited by treatment with SFII at a concentration of 25 μg/mL. Taken together, SFII not only inhibits TNF-α/IFN-γ-induced TARC and MDC but also inhibits CTSS.

### 2.2. TNF-α/IFN-γ-Induced TARC, MDC, and CTSS Expression Is Mediated by STAT1, NF-κB, and p38 MAPK Activation

**Figure 3:**
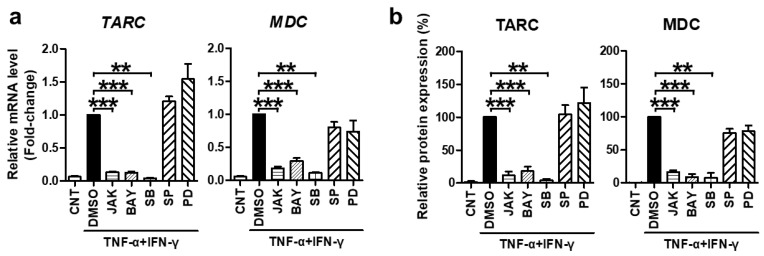
TNF-α/IFN-γ-induced TARC and MDC expression were mediated by STAT1, NF-κB, and p38 MAPK. Cells were pretreated with the JAK I inhibitor (JAK, 5 μM), BAY 11-7082 (BAY, 25 μM), SB203580 (SB, 40 μM), SP600125 (SP, 20 μM), PD98059 (PD, 20 μM), or DMSO for 30 min and then stimulated with TNF-α/IFN-γ for 18 h (for mRNA) or 24 h (for protein). (**a**) The expression of *TARC* and *MDC* mRNA was measured by quantitative real-time PCR. (**b**) Protein levels of TARC and MDC were measured using ELISA with the conditioned media. The mRNA and protein levels, relative to those in the TNF-α/IFN-γ-stimulated DMSO control samples, are presented as mean fold-changes ± SEM of independent experiments (N = 3 or 4). Statistical comparisons were performed using a one-way ANOVA followed by Bonferroni’s post-hoc test (** *p* < 0.01, *** *p* < 0.001).

Subsequently, we investigated the molecular mechanism underlying the suppressive effect of SFII on TNF-α/IFN-γ-stimulated TARC, MDC, and CTSS in HaCaT cells. TNF-α and IFN-γ are known to induce chemokine production by activating STAT1, NF-κB, and p38 [33]. Moreover, *TARC* and *MDC* promoters contain these transcription factor-binding sequences [34]. In this context, many studies have shown that STAT1, NF-κB, and p38 are mediators of TNF-α/IFN-γ-induced TARC and MDC production [35,36]. However, the mediator of CTSS is not well known. Therefore, we pre-treated cells with specific inhibitors for 30 min before TNF-α/IFN-γ-stimulation to identify signaling molecules that mediate the expression of TARC and MDC and to find new signaling transcription factors that mediate CTSS expression. The inhibitors of STAT1 (JAK inhibitor I), NF-κB p65 (BAY11-7082), and p38 MAPK (SB203580) significantly inhibited TNF-α/IFN-γ-induced mRNA and protein expression of TARC and MDC, whereas JNK (SP600125) and ERK (PD98059) inhibitors did not (Figure 3).

**Figure 4:**
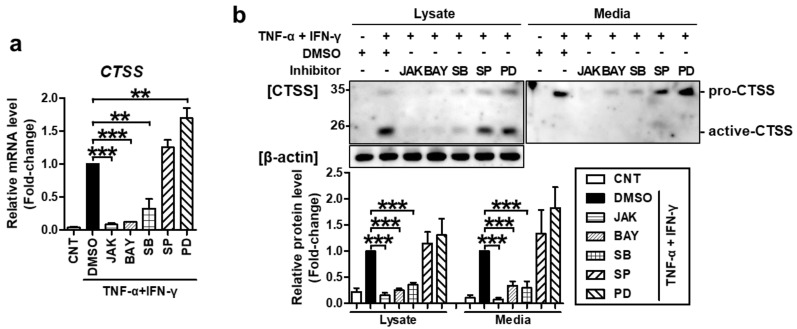
TNF-α/IFN-γ-induced CTSS expression was mediated by STAT1, NF-κB, and p38 MAPK. Cells were treated as in Figure 3. (**a**) The expression of *CTSS* mRNA was measured by quantitative real-time PCR. (**b**) Protein levels of CTSS in cell lysates or conditioned media were measured by western blotting. Band intensity was analyzed using ImageJ software and the combined sum of pro and active CTSS band intensities was normalized to that of β-actin. The mRNA and protein levels, relative to those in the TNF-α/IFN-γ-stimulated DMSO control samples, are presented as mean fold-changes ± SEM of independent experiments (N = 3 or 4). Statistical comparisons were performed using a one-way ANOVA followed by Bonferroni’s post-hoc test (** *p* < 0.01, *** *p* < 0.001).

Furthermore, the same inhibitors also suppressed TNF-α/IFN-γ-induced CTSS mRNA and protein expression (Figure 4). These results confirmed that STAT1, NF-κB, and p38 MAPK mediate TNF-α/IFN-γ-induced TARC, MDC, and CTSS production.

### 2.3. SFII Suppresses TARC, MDC, and CTSS by Inhibiting TNF-α/IFN-γ-Induced Phosphorylation of STAT1, p65, and p38 MAPK

**Figure 5:**
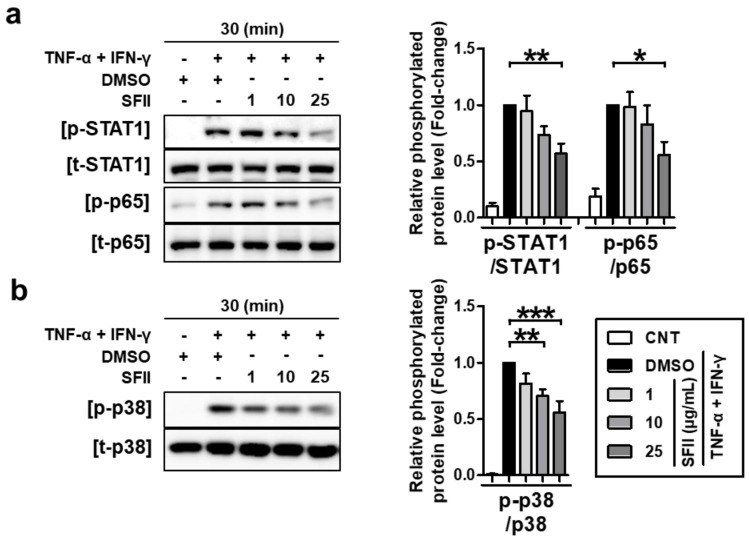
SFII suppressed TNF-α/IFN-γ-induced TARC, MDC, and CTSS by inhibiting STAT1, p65, and p38 MAPK activation. Cells were pretreated with 1, 10, and 25 μg/mL of SFII or DMSO, 30 min prior to TNF-α/IFN-γ-stimulation, and then, the cell lysate samples were harvested at 30 min after stimulation. Protein levels of (**a**) phosphorylated and total forms of STAT1 (p/t-STAT1), p65 (p/t-p65), and (**b**) p38 (p/t-p38) were measured by western blotting. Band intensity was analyzed using ImageJ software and normalized to the total form of each protein. Relative phosphorylated protein levels, normalized to those in the TNF-α/IFN-γ-stimulated DMSO control sample, are presented as the mean fold-change ± SEM of data (N = 4). Statistical comparisons were performed using a one-way ANOVA followed by Bonferroni’s post-hoc test (* *p* < 0.05, ** *p* < 0.01, *** *p* < 0.001).

We next examined the effects of SFII on TNF-α/IFN-γ-induced phosphorylation of STAT1, p65, and p38 MAPK. HaCaT cells were pretreated with the indicated concentrations of SFII for 30 min and stimulated with TNF-α/IFN-γ for 30 min, and then, lysates were harvested. As shown in Figure 5a, SFII inhibited the phosphorylation of STAT1 (p-STAT1) and p65 (p-p65 NF-κB) induced by TNF-α/IFN-γ. The densitometry results of the phosphorylation form versus total form revealed that SFII significantly suppressed both p-STAT1 and p-p65 at 25 μg/mL.

We then investigated the effect of SFII on the p38 MAPK kinase, which is an upstream mediator of STAT1 and NF-κB signaling in TNF-α/IFN-γ-stimulation [36]. SFII showed a significant suppressive effect on p38 activation from 10 to 25 μg/mL in a dose-dependent manner (Figure 5b). These results suggest that SFII suppresses TNF-α/IFN-γ-induced phosphorylation of STAT1, p65, and p38 MAPK, resulting in the suppression of TARC, MDC, and CTSS.

## 3. Discussion

*S. baicalensis* is widely used in traditional medicine, and its main bioactive flavonoids, baicalin and baicalein, have been found to have diverse beneficial activities, including antioxidant and anti-inflammatory effects [37,38]. They are being extensively studied for the treatment of various metabolic syndromes, respiratory diseases, autoimmune diseases, cancers, and inflammatory diseases in different organs [37,38]. Root extracts of *S. baicalensis* and its major components, baicalin and baicalein, have also been reported to exhibit anti-inflammatory effects on AD-induced NC/Nga mice [39,40].

SFII is another bioactive flavonoid compound found in the root extracts of *S. baicalensis* [41]. It is also known as neobaicalein, due to its structural similarity to baicalein [42]; however, SFII is not as actively studied, probably owing to its relatively low abundance in *S. baicalensis* extracts [43]. Nevertheless, a few previous studies have reported the anti-inflammatory activity of SFII. SFII inhibited Th2 cytokine production by regulating NF-κB signaling in allergic rhinitis [26] and attenuated the inflammatory responses in an ovalbumin-induced allergic asthma mouse model by regulating TGF-β1/Smad signaling pathways [30]. In another study, SFII was found to inhibit FBS- or TNF-α-induced MMP-1 expression by regulating ERK/JNK and NF-κB signaling pathways, respectively, in human dermal fibroblasts [32]. In our study, we demonstrated for the first time that SFII can regulate JAK/STAT1 and p38 MAPK pathways, and also inhibits the NF-κB pathway, all of which are well-known pathways involved in the TNF-α/IFN-γ-induced inflammatory response model using HaCaT cells [35,36].

The range of SFII concentrations employed has varied widely across studies, due to its differential range of cytotoxicity according to cell types. For instance, SFII concentrations more than 2 μM were toxic to bone marrow-derived macrophages [31], but no cytotoxicity was observed till 10 μM in human dermal fibroblasts [32], and at 20–250 μM in SH-SY5Y neuroblastoma cells [42]. Similar to SH-SY5Y cells, a SFII concentration range of 1–25 μg/mL (=2.7–66.7 μM) was used in the current study in HaCaT cells, with no cytotoxicity.

NF-κB and p38 MAPK are thought to be key regulators of inflammatory diseases [44,45]. Moreover, p38 is known to enhance NF-κB signaling [46]. In usual circumstances, NF-κB exists in the cytosol in an inactive form complexed with inhibitors of κB (IκB). Phosphorylation and degradation of IκB are induced in response to cellular stimuli, resulting in the release of free activated NF-κB heterodimers, followed by their translocation into the nucleus, with phosphorylation on the p65 subunit-containing transactivation domain [44]. TNF-α is one of the most well-known activators of the NF-κB pathway [44]. In many cell types, TNF-α induces the degradation of IkBα, and the phosphorylation and nuclear translocation of p65 [44]. p38 MAP kinases (p38α, p38β, p38γ, and p38δ) are serine/threonine protein kinases that play roles in responses to various external stress signals [45]. p38α, the most studied kinase, is known to participate in inflammatory signal transduction and cytokine production [45]. p38 has also been reported to contribute to the expression of TARC and MDC induced by TNF-α/IFN-γ in HaCaT cells, as an upstream regulator of STAT1 and the NF-κB pathway [36]. Inhibition of p38 was observed to downregulate phosphorylation of STAT1 and nuclear translocation of p65 [36]. In addition, p38 was also found to regulate the phosphorylation and phosphoacetylation of histone H3, enhancing the recruitment of NF-κB to binding sites in the H3 phosphorylated area [46], independent of p65 translocation. Therefore, inhibition of both NF-κB and the p38 MAPK pathways by SFII as demonstrated in the current study could have a synergistic regulatory effects on NF-κB-mediated inflammatory gene expression. However, the inhibition of p65 and p38 phosphorylation by SFII was less pronounced than that of STAT1. A combination of SFII with stronger antagonists of the NF-κB or p38 MAPK pathway might confer stronger anti-inflammatory effects.

STAT proteins are well known for their roles in transducing cytokine-mediated signals and specifying Th cell differentiation [47]. There are seven members of the STAT family, and it is generally known that the development of Th1, Th2, and Th17 immune responses is mediated by STAT4/STAT1, STAT6/STAT5, and STAT3, respectively [48]. Among them, STAT1 is responsible for the transduction of type 1 IFN signals (α and β) and type 2 signals (γ) through a JAK1/2-dependent mechanism [49]. In particular, it is a major signal transmitter of IFN-γ, an important mediator of immune responses and inflammation [50,51], and its deficiency results in susceptibility to viral diseases and impaired responses to IFN-γ and IL-27 [47,52,53]. Some reports postulate a role for STAT1 in terms of the Th2-immune response in AD. In addition to mediating TARC and MDC production by TNF-α/IFN-γ-stimulation in HaCaT cells [54,55], it has been reported that STAT1 can be activated by the human TSLP receptor [48,56]. Moreover, IL-31 which is mainly produced by Th2 cells, activates JAK1 and/or JAK2 and, STAT1, STAT3, and STAT5 [4]. Since TSLP and IL-31 mediate the histamine-independent itch pathway, STAT1 might also be involved in pruritus in AD [4]. In this context, JAK/STAT signaling is considered as one of the representative therapeutic targets of several inflammatory diseases, and recently, JAK inhibitors have been applied as a new therapeutic strategy for AD [7,57]. Thus, the inhibition of STAT1 phosphorylation in TNF-α/IFN-γ-stimulated HaCaT cells by SFII, and therefore, it has a potential therapeutic effect on AD.

Moreover, we revealed for the first time that the regulation of TNF-α/IFN-γ-induced CTSS production (in addition to TARC and MDC) is also mediated by STAT1, NF-κB, and p38 MAPK pathways. We also confirmed that ERK and JNK are not involved in TNF-α/IFN-γ-induced cytokine or CTSS expression, similar to earlier reports [17,58]. CTSS is known to activate PAR2 signaling [20], a main mediator of itching signals in AD [21], and CTSS-overexpressing transgenic mice have been reported to develop a chronic AD-like skin phenotype [24]. PAR2 signaling activates histamine-independent itching pathways, like the release of pruritogenic mediators and increased expression of TSLP, and histamine antagonists are known to be less effective as treatment for atopic dermatitis-related itch. In such conditions, the regulatory effect of SFII on CTSS confer an advantage in AD-related itch treatment.

Because TARC and MDC are well-known Th2 chemokines, highly expressed in skin lesions and plasma of patients with AD [9,10,54], the inhibitory activity of SFII against TNF-α/IFN-γ-induced TARC and MDC expression suggests its possible anti-inflammatory effects on AD. In several studies, molecules or plant extracts that exhibited inhibitory effects on TARC and MDC induction in TNF-α/IFN-γ-stimulated HaCaT cells also showed anti-inflammatory effects in AD-like mouse models induced by various reagents, such as 2,4-dinitrochlorobenzene (DNCB) or 1-fluoro-2,4-dinitrobenzene (DNFB) [59,60,61].

We could not examine the effects of SFII on other cytokines important for the development of AD, such as IL-25, 33, or TSLP. A few reports have discussed the induction of these cytokines in TNF-α/IFN-γ-stimulated HaCaT cells [62,63,64]; however, in our experiments, mRNA expression of IL-25, IL-33, and TSLP was either not detected or detected at very low levels. While this difference might be attributable to the experimental conditions, it is also possible that TNF-α/IFN-γ-stimulated HaCaT might not have been a suitable model for investigation of the effects of SFII on the expression of IL-25, IL-33, or TSLP. In addition, we also attempted TNF-α/IFN-γ stimulation on primary keratinocytes; however, we could not detect any secretion of TARC in the conditioned medium using ELISA. To our knowledge, for TNF-α/IFN-γ stimulation experiments, nearly all studies were performed using HaCaT cells, and studies using primary keratinocytes were scarce. We found one study that included the TNFα and IFN-γ-mediated inductions of TARC and MDC in primary keratinocytes [10]. In that study, the authors revealed that TNFα and IFN-γ, or only IFN-γ treatment, could induce *TARC* and *MDC* mRNA expressions both in primary keratinocytes and HaCaT cells. However, the secretion of TARC by stimulation with IFN-γ in the conditioned medium of primary keratinocytes was not detected using ELISA, while that of MDC was detected. We still do not know the reason for this phenomenon, but it seems to be the reason why HaCaT cells are generally used for this experiment.

In conclusion, we newly found that SFII inhibits TNF-α/IFN-γ-induced TARC, MDC, and CTSS expression by regulating STAT1, NF-κB, and p38 MAPK signaling pathways. Our findings imply that SFII might be a potential therapeutic agent for inflammatory diseases, such as AD. Additional preclinical and clinical studies are warranted to validate the anti-inflammatory activity of SFII.

## 4. Materials and Methods

### 4.1. Antibodies and Reagents

SFII was obtained from the Korea Research Institute of Bioscience and Biotechnology (KRIBB, Daejeon, South Korea) [30]. Recombinant TNF-α and IFN-γ were purchased from R&D Systems (Minneapolis, MN, USA). Primary antibodies against p-STAT1 (sc-592, 1:5000), t-STAT1 (sc-7988, 1:2000), and CTSS (sc-6503, 1:1000) were obtained from Santa Cruz Biotechnology Inc. (Santa Cruz, CA, USA), and those against p-p65 (CST3031S, 1:6000), t-p65 (CST8242S, 1:2000), p-p38 (CST9211S, 1:2000), and t-p38 (CST9212S, 1:2000) were from Cell Signaling Technology Inc. (Danvers, MA, USA). Primary antibodies against β-actin (Thermo Fisher Scientific, Waltham, MA, USA, MA5-15739, 1:2000) and horseradish peroxidase-conjugated polyclonal secondary antibodies against mouse, rabbit, or goat IgG (GeneTex, Inc., Irvine, CA, USA, 1:10,000) were also purchased. The inhibitors SB203580 (p38 inhibitor), SP600125 (JNK inhibitor), and PD98059 (MEK1 inhibitor) were purchased from Tocris (Bristol, UK); InSolution™ JAK inhibitor I and BAY 11-7082 (NF-κB inhibitor) were purchased from Calbiochem (San Diego, CA, USA).

### 4.2. Cell Culture

HaCaT cells, an immortalized human keratinocyte cell line, were kindly provided by Dr. N.E. Fusenig, DKFZ, Heidelberg, Germany [65]. They were cultured in Dulbecco’s modified Eagle’s medium (DMEM; Welgene, Daegu, Korea) supplemented with 10% fetal bovine serum (FBS; Gibco, Rockville, MD, USA) and 1% penicillin–streptomycin (Gibco), in a humidified 5% CO_2_ atmosphere at 37 °C. For the TNF-α/IFN-γ-stimulation model, cells were first seeded (1.5 × 10^5^ cells/dish in a 35 mm dish) and grown for 2–3 days and then starved with 0% FBS-DMEM for 1 day at >90% confluence. Cells were then treated with SFII, inhibitors, or DMSO in 0% FBS-DMEM for 30 min and then co-stimulated with TNF-α (2 ng/mL) and IFN-γ (10 ng/mL) for the indicated times. In the lactate dehydrogenase activity (LDH) assay, the cells were incubated without cytokine treatment.

### 4.3. Cell Cytotoxicity

Cytotoxicity induced by treatment with SFII was determined using the EZ-LDH Cell Cytotoxicity Assay Kit (DoGenBio, Seoul, Korea). Briefly, cells were seeded in two sets (cytotoxicity and control groups) and treated with the indicated concentrations of SFII. At 24 h after SFII treatment, water soluble tetrazolium salt (WST) was added to both groups, and lysis buffer was added to the control group, followed by color development and measurement of absorbance at 450 nm. The proportion of dead cells was calculated from the ratio of absorbance in each group.

### 4.4. Enzyme-Linked Immunosorbent Assay (ELISA)

Supernatants were collected from cells after 24 h of TNF-α/IFN-γ stimulation. TARC and MDC production was then measured by ELISA using DuoSet ELISA kits for human TARC (DY364) and MDC (DY336) (R&D Systems), according to the manufacturer’s instructions.

### 4.5. Quantitative Real-Time PCR

Total RNA was isolated from cultured HaCaT cells using RNAiso Plus (Takara Bio Inc., Shiga, Japan) at 18 h after TNF-α/IFN-γ-stimulation. Subsequent cDNA synthesis and product confirmation by real-time PCR was performed as detailed in our previous report [66]. The following primer sequences were used for amplification: *TARC* forward: 5′-TTGTAACTGTGCAGGGCAGG-3′, reverse: 5′-TGAACACCAACGGTGGAGGT-3′, *MDC* forward: 5′-GAAGCCTGTGCCAACTCTCT-3′, reverse: 5′-GGGAATCGCTGATGGGAACA-3′, *CTSS* forward: 5′-TGGGCTTTCAGTGCTGTGGG-3′, reverse: 5′-TCAATGATGTACTGGAAAGC-3′, *18S rRNA* forward: 5′-GTAACCCGTTGAACCCCATT-3′, reverse: 5′-CCATCCAATCGGTAGTAGCG-3′, *GAPDH* forward: 5′-ATTGTTGCCATCAATGACCC-3′, reverse: 5′-AGTAGAGGCAGGGATGATGT-3′, *U6 snRNA* forward: 5′-CTCGCTTCGGCAGCACA-3′, and reverse: 5′-AACGCTTCACGAATTTGCGT-3′. The data were analyzed by the 2^(−∆∆Ct)^ method and are represented as fold-changes of gene expression, relative to levels in the group of TNF-α/IFN-γ-stimulated cells pretreated with DMSO; the expression was normalized to that of three endogenous control genes, *18S rRNA*, *GAPDH*, and *U6 snRNA*.

### 4.6. Western Blot Analysis

After TNF-α/IFN-γ-stimulation, cell lysates were harvested at 30 min, and cell lysates or conditioned media for CTSS analysis were collected at 24 h to determine the expression of signaling molecules. To extract the proteins for western blotting, cells were harvested by scraping on ice with 1× SDS-lysis buffer containing high concentrations of SDS (1.75%) and 2-mercaptoethanol (715 mM), along with the Complete Mini protease inhibitor cocktail (Roche Applied Science, Indianapolis, IN, USA) and phosphatase inhibitor cocktails (Sigma-Aldrich, St. Louis, MO, USA). Subsequent processes were performed as detailed in ourprevious report [66]. Membranes were blocked using 5% BSA in tris-buffered saline with 0.1% Tween^®^ 20 (TBS-T), instead of 5% skim milk in TBS-T for the phospho-forms of target proteins. A CCD camera, Amersham Imager 680 (GE Healthcare, Chicago, IL, USA) was used for enhanced chemiluminescence signal detection. Only data with unsaturated signals were used for analysis.

### 4.7. Statistical Analysis

Statistical analyses were performed using GraphPad Prism software v.5.0.3 (GraphPad Prism, La Jolla, CA, USA). A one-way ANOVA followed by Bonferroni’s post-hoc test was used to compare the groups. Data are presented as the mean ± SEM of three or more independent experiments. Statistical significance was set at *p* < 0.05.

## Abbreviations

SFII	Skullcapflavone II
TNF-α	tumor necrosis factor-α
IFN-γ	interferon-γ
TARC	thymus- and activation-regulated chemokine
MDC	macrophage-derived chemokine
CTSS	cathepsin S
AD	atopic dermatitis

## References

[1] Langan S.M., Irvine A.D., Weidinger S. (2020). Atopic dermatitis. Lancet.

[2] Nakahara T., Kido-Nakahara M., Tsuji G., Furue M. (2021). Basics and recent advances in the pathophysiology of atopic dermatitis. J. Dermatol..

[3] Leung D.Y., Guttman-Yassky E. (2014). Deciphering the complexities of atopic dermatitis: Shifting paradigms in treatment approaches. J. Allergy Clin. Immunol..

[4] Weidinger S., Beck L.A., Bieber T., Kabashima K., Irvine A.D. (2018). Atopic dermatitis. Nat. Rev. Dis. Prim..

[5] Imai T., Nagira M., Takagi S., Kakizaki M., Nishimura M., Wang J., Gray P.W., Matsushima K., Yoshie O. (1999). Selective recruitment of CCR4-bearing Th2 cells toward antigen-presenting cells by the CC chemokines thymus and activation-regulated chemokine and macrophage-derived chemokine. Int. Immunol..

[6] Kataoka Y. (2014). Thymus and activation-regulated chemokine as a clinical biomarker in atopic dermatitis. J. Dermatol..

[7] Stander S. (2021). Atopic Dermatitis. N. Engl. J. Med..

[8] Jaworek A.K., Szafraniec K., Zuber Z., Wojas-Pelc A., Jaworek J. (2020). Interleukin 25, thymic stromal lymphopoietin and house dust mites in pathogenesis of atopic dermatitis. J. Physiol. Pharmacol..

[9] Kakinuma T., Nakamura K., Wakugawa M., Mitsui H., Tada Y., Saeki H., Torii H., Asahina A., Onai N., Matsushima K. (2001). Thymus and activation-regulated chemokine in atopic dermatitis: Serum thymus and activation-regulated chemokine level is closely related with disease activity. J. Allergy Clin. Immunol..

[10] Horikawa T., Nakayama T., Hikita I., Yamada H., Fujisawa R., Bito T., Harada S., Fukunaga A., Chantry D., Gray P.W. (2002). IFN-gamma-inducible expression of thymus and activation-regulated chemokine/CCL17 and macrophage-derived chemokine/CCL22 in epidermal keratinocytes and their roles in atopic dermatitis. Int. Immunol..

[11] Thijs J., Krastev T., Weidinger S., Buckens C.F., de Bruin-Weller M., Bruijnzeel-Koomen C., Flohr C., Hijnen D. (2015). Biomarkers for atopic dermatitis: A systematic review and meta-analysis. Curr. Opin. Allergy Clin. Immunol..

[12] Nedoszytko B., Sokolowska-Wojdylo M., Ruckemann-Dziurdzinska K., Roszkiewicz J., Nowicki R.J. (2014). Chemokines and cytokines network in the pathogenesis of the inflammatory skin diseases: Atopic dermatitis, psoriasis and skin mastocytosis. Postep. Dermatol. Alergol..

[13] Brandt E.B., Sivaprasad U. (2011). Th2 Cytokines and Atopic Dermatitis. J. Clin. Cell. Immunol..

[14] Vestergaard C., Yoneyama H., Murai M., Nakamura K., Tamaki K., Terashima Y., Imai T., Yoshie O., Irimura T., Mizutani H. (1999). Overproduction of Th2-specific chemokines in NC/Nga mice exhibiting atopic dermatitis-like lesions. J. Clin. Investig..

[15] Vestergaard C., Bang K., Gesser B., Yoneyama H., Matsushima K., Larsen C.G. (2000). A Th-2 chemokine, TARC, produced by keratinocytes may recruit CLA(+)CCR4(+) lymphocytes into lesional atopic dermatitis skin. J. Investig. Dermatol..

[16] Yano C., Saeki H., Komine M., Kagami S., Tsunemi Y., Ohtsuki M., Nakagawa H. (2015). Mechanism of Macrophage-Derived Chemokine/CCL22 Production by HaCaT Keratinocytes. Ann. Dermatol..

[17] Komine M., Kakinuma T., Kagami S., Hanakawa Y., Hashimoto K., Tamaki K. (2005). Mechanism of thymus- and activation-regulated chemokine (TARC)/CCL17 production and its modulation by roxithromycin. J. Investig. Dermatol..

[18] Jia J., Mo X., Yan F., Liu J., Ye S., Zhang Y., Lin Y., Li H., Chen D. (2021). Role of YAP-related T cell imbalance and epidermal keratinocyte dysfunction in the pathogenesis of atopic dermatitis. J. Dermatol. Sci..

[19] Kim S.M., Ha S.E., Vetrivel P., Kim H.H., Bhosale P.B., Park J.E., Heo J.D., Kim Y.S., Kim G.S. (2020). Cellular Function of Annexin A1 Protein Mimetic Peptide Ac2-26 in Human Skin Keratinocytes HaCaT and Fibroblast Detroit 551 Cells. Nutrients.

[20] Reddy V.B., Shimada S.G., Sikand P., LaMotte R.H., Lerner E.A. (2010). Cathepsin S Elicits Itch and Signals via Protease-Activated Receptors. J. Investig. Dermatol..

[21] Steinhoff M., Neisius U., Ikoma A., Fartasch M., Heyer G., Skov P.S., Luger T.A., Schmelz M. (2003). Proteinase-activated receptor-2 mediates itch: A novel pathway for pruritus in human skin. J. Neurosci..

[22] Schwarz G., Boehncke W.H., Braun M., Schroter C.J., Burster T., Flad T., Dressel D., Weber E., Schmid H., Kalbacher H. (2002). Cathepsin S activity is detectable in human keratinocytes and is selectively upregulated upon stimulation with interferon-gamma. J. Investig. Dermatol..

[23] Schonefuss A., Wendt W., Schattling B., Schulten R., Hoffmann K., Stuecker M., Tigges C., Lubbert H., Stichel C. (2010). Upregulation of cathepsin S in psoriatic keratinocytes. Exp. Dermatol..

[24] Kim N., Bae K.B., Kim M.O., Yu D.H., Kim H.J., Yuh H.S., Ji Y.R., Park S.J., Kim S., Son K.H. (2012). Overexpression of cathepsin S induces chronic atopic dermatitis in mice. J. Investig. Dermatol..

[25] Ishimaru K., Nishikawa K., Omoto T., Asai I., Yoshihira K., Shimomura K. (1995). Two flavone 2’-glucosides from Scutellaria baicalensis. Phytochemistry.

[26] Bui T.T., Piao C.H., Song C.H., Chai O.H. (2017). Skullcapflavone II attenuates ovalbumin-induced allergic rhinitis through the blocking of Th2 cytokine production and mast cell histamine release. Int. Immunopharmacol..

[27] Salaritabar A., Darvishi B., Hadjiakhoondi F., Manayi A., Sureda A., Nabavi S.F., Fitzpatrick L.R., Nabavi S.M., Bishayee A. (2017). Therapeutic potential of flavonoids in inflammatory bowel disease: A comprehensive review. World J. Gastroenterol..

[28] Abotaleb M., Samuel S.M., Varghese E., Varghese S., Kubatka P., Liskova A., Busselberg D. (2018). Flavonoids in Cancer and Apoptosis. Cancers.

[29] Tsai P.J., Huang W.C., Hsieh M.C., Sung P.J., Kuo Y.H., Wu W.H. (2015). Flavones Isolated from Scutellariae radix Suppress Propionibacterium Acnes-Induced Cytokine Production In Vitro and In Vivo. Molecules.

[30] Jang H.Y., Ahn K.S., Park M.J., Kwon O.K., Lee H.K., Oh S.R. (2012). Skullcapflavone II inhibits ovalbumin-induced airway inflammation in a mouse model of asthma. Int. Immunopharmacol..

[31] Lee J., Son H.S., Lee H.I., Lee G.R., Jo Y.J., Hong S.E., Kim N., Kwon M., Kim N.Y., Kim H.J. (2019). Skullcapflavone II inhibits osteoclastogenesis by regulating reactive oxygen species and attenuates the survival and resorption function of osteoclasts by modulating integrin signaling. FASEB J..

[32] Lee Y.H., Seo E.K., Lee S.T. (2019). Skullcapflavone II Inhibits Degradation of Type I Collagen by Suppressing MMP-1 Transcription in Human Skin Fibroblasts. Int. J. Mol. Sci..

[33] Ju S.M., Song H.Y., Lee S.J., Seo W.Y., Sin D.H., Goh A.R., Kang Y.H., Kang I.J., Won M.H., Yi J.S. (2009). Suppression of thymus- and activation-regulated chemokine (TARC/CCL17) production by 1,2,3,4,6-penta-O-galloyl-beta-D-glucose via blockade of NF-kappaB and STAT1 activation in the HaCaT cells. Biochem. Biophys. Res. Commun..

[34] Nakayama T., Hieshima K., Nagakubo D., Sato E., Nakayama M., Kawa K., Yoshie O. (2004). Selective induction of Th2-attracting chemokines CCL17 and CCL22 in human B cells by latent membrane protein 1 of Epstein-Barr virus. J. Virol..

[35] Park J.W., Lee H.S., Lim Y., Paik J.H., Kwon O.K., Kim J.H., Paryanto I., Yunianto P., Choi S., Oh S.R. (2018). Rhododendron album Blume extract inhibits TNF-/IFN--induced chemokine production via blockade of NF-B and JAK/STAT activation in human epidermal keratinocytes. Int. J. Mol. Med..

[36] Kwon D.J., Bae Y.S., Ju S.M., Goh A.R., Youn G.S., Choi S.Y., Park J. (2012). Casuarinin suppresses TARC/CCL17 and MDC/CCL22 production via blockade of NF-kappaB and STAT1 activation in HaCaT cells. Biochem. Biophys. Res. Commun..

[37] Baradaran Rahimi V., Askari V.R., Hosseinzadeh H. (2021). Promising influences of Scutellaria baicalensis and its two active constituents, baicalin, and baicalein, against metabolic syndrome: A review. Phytother. Res. PTR.

[38] Dinda B., Dinda S., DasSharma S., Banik R., Chakraborty A., Dinda M. (2017). Therapeutic potentials of baicalin and its aglycone, baicalein against inflammatory disorders. Eur. J. Med. Chem.

[39] Yun M.Y., Yang J.H., Kim D.K., Cheong K.J., Song H.H., Kim D.H., Cheong K.J., Kim Y.I., Shin S.C. (2010). Therapeutic effects of Baicalein on atopic dermatitis-like skin lesions of NC/Nga mice induced by dermatophagoides pteronyssinus. Int. Immunopharmacol..

[40] Kim J., Lee I., Park S., Choue R. (2010). Effects of Scutellariae radix and Aloe vera gel extracts on immunoglobulin E and cytokine levels in atopic dermatitis NC/Nga mice. J. Ethnopharmacol..

[41] Kubo M., Matsuda H., Tanaka M., Kimura Y., Okuda H., Higashino M., Tani T., Namba K., Arichi S. (1984). Studies on Scutellariae radix. VII. Anti-arthritic and anti-inflammatory actions of methanolic extract and flavonoid components from Scutellariae radix. Chem. Pharm. Bull..

[42] Parsafar S., Nayeri Z., Aliakbari F., Shahi F., Mohammadi M., Morshedi D. (2020). Multiple neuroprotective features of Scutellaria pinnatifida-derived small molecule. Heliyon.

[43] Wang Z.L., Wang S., Kuang Y., Hu Z.M., Qiao X., Ye M. (2018). A comprehensive review on phytochemistry, pharmacology, and flavonoid biosynthesis of Scutellaria baicalensis. Pharm. Biol..

[44] Tak P.P., Firestein G.S. (2001). NF-kappa B: A key role in inflammatory diseases. J. Clin. Investig..

[45] Kumar S., Boehm J., Lee J.C. (2003). p38 map kinases: Key signalling molecules as therapeutic targets for inflammatory diseases. Nat. Rev. Drug Discov..

[46] Saccani S., Pantano S., Natoli G. (2002). p38-dependent marking of inflammatory genes for increased NF-kappa B recruitment. Nat. Immunol..

[47] O’Shea J.J., Lahesmaa R., Vahedi G., Laurence A., Kanno Y. (2011). Genomic views of STAT function in CD4+ T helper cell differentiation. Nat. Rev. Immunol..

[48] Bao L., Zhang H., Chan L.S. (2013). The involvement of the JAK-STAT signaling pathway in chronic inflammatory skin disease atopic dermatitis. JAK-STAT.

[49] Garcia-Melendo C., Cubiro X., Puig L. (2021). Janus Kinase Inhibitors in Dermatology: Part 2: Applications in Psoriasis, Atopic Dermatitis, and Other Dermatoses. Actas Dermosifiliogr..

[50] Hu X., Ivashkiv L.B. (2009). Cross-regulation of signaling pathways by interferon-gamma: Implications for immune responses and autoimmune diseases. Immunity.

[51] Ramana C.V., Gil M.P., Schreiber R.D., Stark G.R. (2002). Stat1-dependent and -independent pathways in IFN-gamma-dependent signaling. Trends Immunol..

[52] Majoros A., Platanitis E., Kernbauer-Holzl E., Rosebrock F., Muller M., Decker T. (2017). Canonical and Non-Canonical Aspects of JAK-STAT Signaling: Lessons from Interferons for Cytokine Responses. Front. Immunol..

[53] Dupuis S., Jouanguy E., Al-Hajjar S., Fieschi C., Al-Mohsen I.Z., Al-Jumaah S., Yang K., Chapgier A., Eidenschenk C., Eid P. (2003). Impaired response to interferon-alpha/beta and lethal viral disease in human STAT1 deficiency. Nat. Genet..

[54] Shimada Y., Takehara K., Sato S. (2004). Both Th2 and Th1 chemokines (TARC/CCL17, MDC/CCL22, and Mig/CXCL9) are elevated in sera from patients with atopic dermatitis. J. Dermatol. Sci..

[55] Thijs J.L., de Bruin-Weller M.S., Hijnen D. (2017). Current and Future Biomarkers in Atopic Dermatitis. Immunol. Allergy Clin. N. Am..

[56] Wohlmann A., Sebastian K., Borowski A., Krause S., Friedrich K. (2010). Signal transduction by the atopy-associated human thymic stromal lymphopoietin (TSLP) receptor depends on Janus kinase function. Biol. Chem..

[57] Damsky W., Peterson D., Ramseier J., Al-Bawardy B., Chun H., Proctor D., Strand V., Flavell R.A., King B. (2021). The emerging role of Janus kinase inhibitors in the treatment of autoimmune and inflammatory diseases. J. Allergy Clin. Immunol..

[58] Qi X.F., Kim D.H., Yoon Y.S., Li J.H., Song S.B., Jin D., Huang X.Z., Teng Y.C., Lee K.J. (2009). The adenylyl cyclase-cAMP system suppresses TARC/CCL17 and MDC/CCL22 production through p38 MAPK and NF-kappaB in HaCaT keratinocytes. Mol. Immunol..

[59] Yang J.H., Do H.J., Lee E., Yim N.H., Cho W.K., Park K.I., Ma J.Y. (2018). Jageum-Jung improves 2,4-dinitrochlorobenzene-induced atopic dermatitis-like skin lesions in mice and suppresses pro-inflammatory chemokine production by inhibiting TNF-alpha/IFN-gamma-induced STAT-1 and NFkappaB signaling in HaCaT cells. J. Ethnopharmacol..

[60] Kee J.Y., Jeon Y.D., Kim D.S., Han Y.H., Park J., Youn D.H., Kim S.J., Ahn K.S., Um J.Y., Hong S.H. (2017). Korean Red Ginseng improves atopic dermatitis-like skin lesions by suppressing expression of proinflammatory cytokines and chemokines in vivo and in vitro. J. Ginseng Res..

[61] Samukawa K., Izumi Y., Shiota M., Nakao T., Osada-Oka M., Miura K., Iwao H. (2012). Red ginseng inhibits scratching behavior associated with atopic dermatitis in experimental animal models. J. Pharmacol. Sci..

[62] Cho S.H., Kim H.S., Lee W., Han E.J., Kim S.Y., Fernando I.P.S., Ahn G., Kim K.N. (2020). Eckol from Ecklonia cava ameliorates TNF-alpha/IFN-gamma-induced inflammatory responses via regulating MAPKs and NF-kappaB signaling pathway in HaCaT cells. Int. Immunopharmacol..

[63] Han E.J., Fernando I.P.S., Kim H.S., Lee D.S., Kim A., Je J.G., Seo M.J., Jee Y.H., Jeon Y.J., Kim S.Y. (2021). (-)-Loliolide Isolated from Sargassum horneri Suppressed Oxidative Stress and Inflammation by Activating Nrf2/HO-1 Signaling in IFN-gamma/TNF-alpha-Stimulated HaCaT Keratinocytes. Antioxidants.

[64] Kim H.J., Baek J., Lee J.R., Roh J.Y., Jung Y. (2018). Optimization of Cytokine Milieu to Reproduce Atopic Dermatitis-related Gene Expression in HaCaT Keratinocyte Cell Line. Immune Netw..

[65] Boukamp P., Petrussevska R.T., Breitkreutz D., Hornung J., Markham A., Fusenig N.E. (1988). Normal keratinization in a spontaneously immortalized aneuploid human keratinocyte cell line. J. Cell Biol..

[66] Lee H., Lim J., Oh J.H., Cho S., Chung J.H. (2021). IGF-1 Upregulates Biglycan and Decorin by Increasing Translation and Reducing ADAMTS5 Expression. Int. J. Mol. Sci..

